# Remdesivir-Induced Liver Injury in a Patient With Coronavirus Disease 2019 and History of Congestive Hepatopathy

**DOI:** 10.7759/cureus.32353

**Published:** 2022-12-09

**Authors:** Georgios P Dividis, Georgios E Papadopoulos, Ioannis Karageorgiou, Georgios Kotronis

**Affiliations:** 1 Department of Internal Medicine, Agios Pavlos General Hospital of Thessaloniki, Thessaloniki, GRC; 2 Department of Cardiology, University Hospital of Ioannina, Ioannina, GRC

**Keywords:** heart failure, sars-cov-2 infection, drug-induced liver injury (dili), congestive hepatopathy, remdesivir

## Abstract

Remdesivir is an antiviral agent used as supportive care in adults with SARS-COV2-induced pneumonia. We report a case of an 81-year-old patient who developed hepatocellular acute liver injury 48 hours after initiating remdesivir. During the investigation, other causes of hepatotoxicity were excluded. A decrease in transaminases and international normalized ratio (INR) was observed 24 hours after cessation of remdesivir. An abdominal CT demonstrated hepatic congestion, retrograde hepatic venous opacification shortly after intravenous contrast injection, and dilatation of hepatic veins and inferior vena cava. We suggest congestive hepatopathy secondary to remdesivir as a possible component of liver injury.

## Introduction

The ongoing pandemic of severe acute respiratory syndrome coronavirus 2 (SARS-CΟV2) infections has led to more than 629 million confirmed cases and over 6.5 million deaths worldwide as of November 2022 [1]. Apart from the pulmonary involvement, direct SARS-COV2-induced liver damage has also been reported [2, 3].

No known effective treatment for COVID-19 has been established. Supportive care remains the mainstay, and numerous candidate agents have been proposed. Remdesivir, an antiviral agent, was superior to placebo in shortening recovery time in adults hospitalized with respiratory tract infections with COVID-19 [4]. On May 1st, 2020, the US Food and Drug Administration issued an emergency use authorization of remdesivir for the treatment of COVID-19.

We report a case of a patient with COVID-19 who developed hepatotoxicity following remdesivir therapy in Thessaloniki, Greece, in 2021.

## Case presentation

An 81-year-old man was admitted with a two-day history of fever, dyspnea, and hypoxia. He has had a past medical history of hypertension, dyslipidemia, chronic obstructive pulmonary disease (COPD) with emphysema, benign prostatic hyperplasia, and depression. His medication included lisinopril, tamsulosin, duloxetine, simvastatin, and inhaled beclomethasone/formoterol. He had been an ex-smoker while an intake of illicit drugs or alcohol was not reported.

On admission, blood pressure was 120/80mmHg, heart rate 80bpm, and body temperature 38 ^o^C. Arterial blood gasses demonstrated partial oxygen pressure of 66,7 mmHg (saturation: 88%) in room air.

On examination, he was alert and orientated, had mild diffuse fine crackles without wheezing, and mild pedal edema, while cardiac auscultation did not reveal definite murmurs. His electrocardiogram demonstrated new-onset atrial fibrillation. Chest X-ray depicted bilateral peripheral opacities. A diagnosis of COVID-19 was confirmed by a positive SARS-COV2 polymerase chain reaction (PCR) test of a nasopharynx specimen.

He started an intravenous five-day remdesivir course (loading dose of 200mg followed by 100mg once a day), oral dexamethasone 6mg per day, and intravenous empirical antibiotic treatment of lower respiratory superinfection with ampicillin/sulbactam. Regarding his new-onset atrial fibrillation, he was started on anticoagulation with subcutaneous enoxaparin 6.000iu twice a day for stroke prevention and metoprolol for rate control resulting soon in sinus rhythm restoration. He received oxygen therapy with a nasal cannula delivering 6lt per minute. He had a steady improvement and remained ambulatory with decreasing oxygen demands.

**Figure 1:**
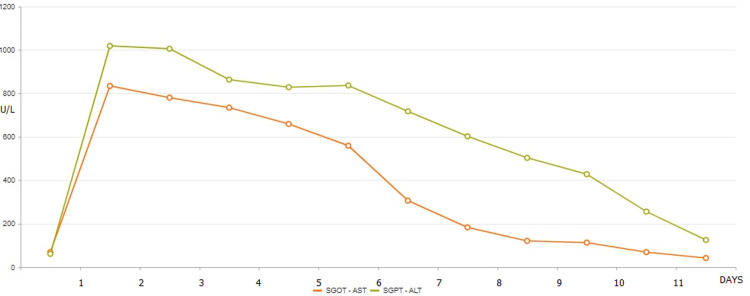
Alanine and aspartate aminotransferases (AST-ALT) concentration vs. time. Y-axis: AST and ALT concentration, units per litre X-axis: days SGOT-AST: serum glutamil oxaloacetic transaminase-aspartate transaminase, SGPT-ALT: serum glutamic pyruvic transaminase-alanine transaminase, U/L: units per litre, ALT: alanine aminotransferase, AST: aspartate aminotransferase

Forty-eight hours later, an acute derangement in liver function tests was noticed, characterized by a predominant hepatocellular liver injury pattern (alanine aminotransferase (ALT) increased from 61 to 1018, aspartate aminotransferase (AST) from 65 to 834; Figure 1) as well as coagulopathy (international normalized ratio (INR) from 0.9 to 1.8). Cholestatic liver enzymes demonstrated mild elevation (alkaline phosphatase from 50 to 96U/L, γ-glutamyltransferase from 28 to 224U/L, total bilirubin 0,9μmol/L).

**Figure 2:**
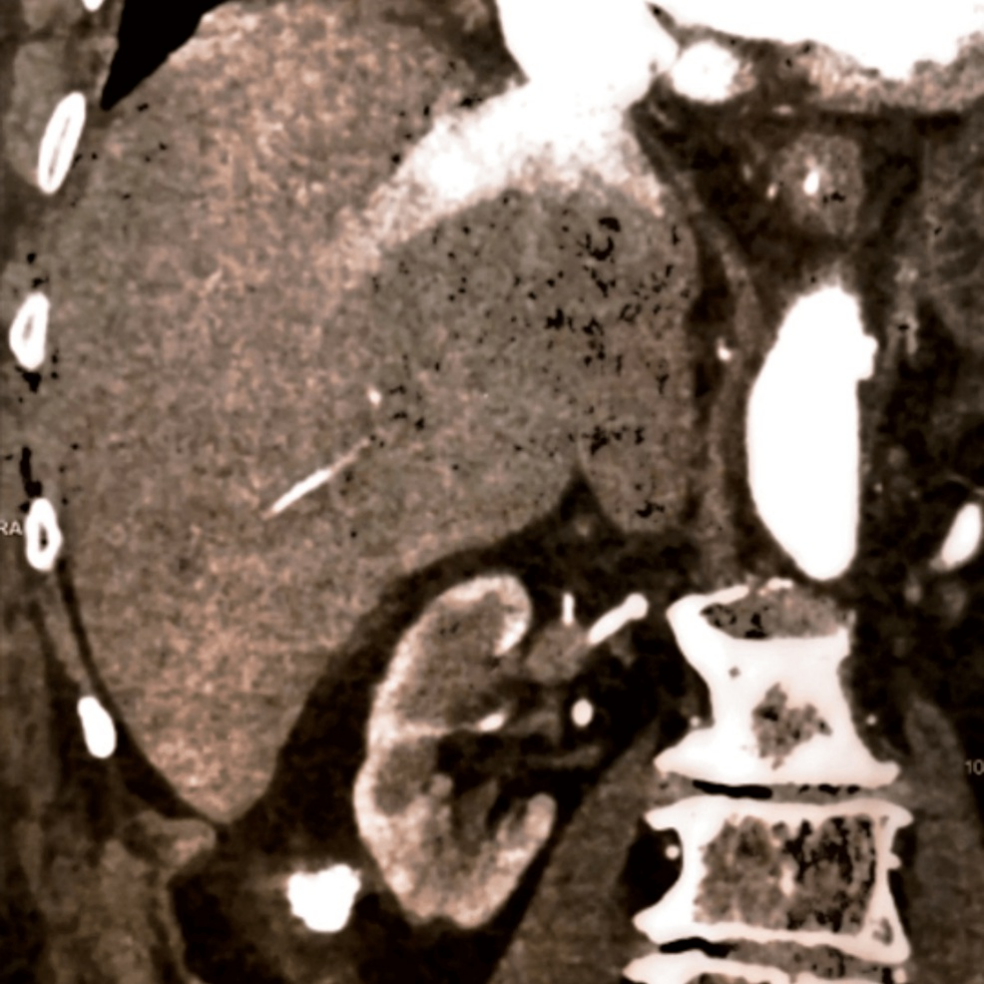
Computed tomography demonstrating mild liver enlargement, inferior vena cava dilatation, hepatic vein dilatation, and cardiac chamber enlargement. Retrograde hepatic venous opacification was depicted shortly after intravenous contrast injection.

**Figure 3:**
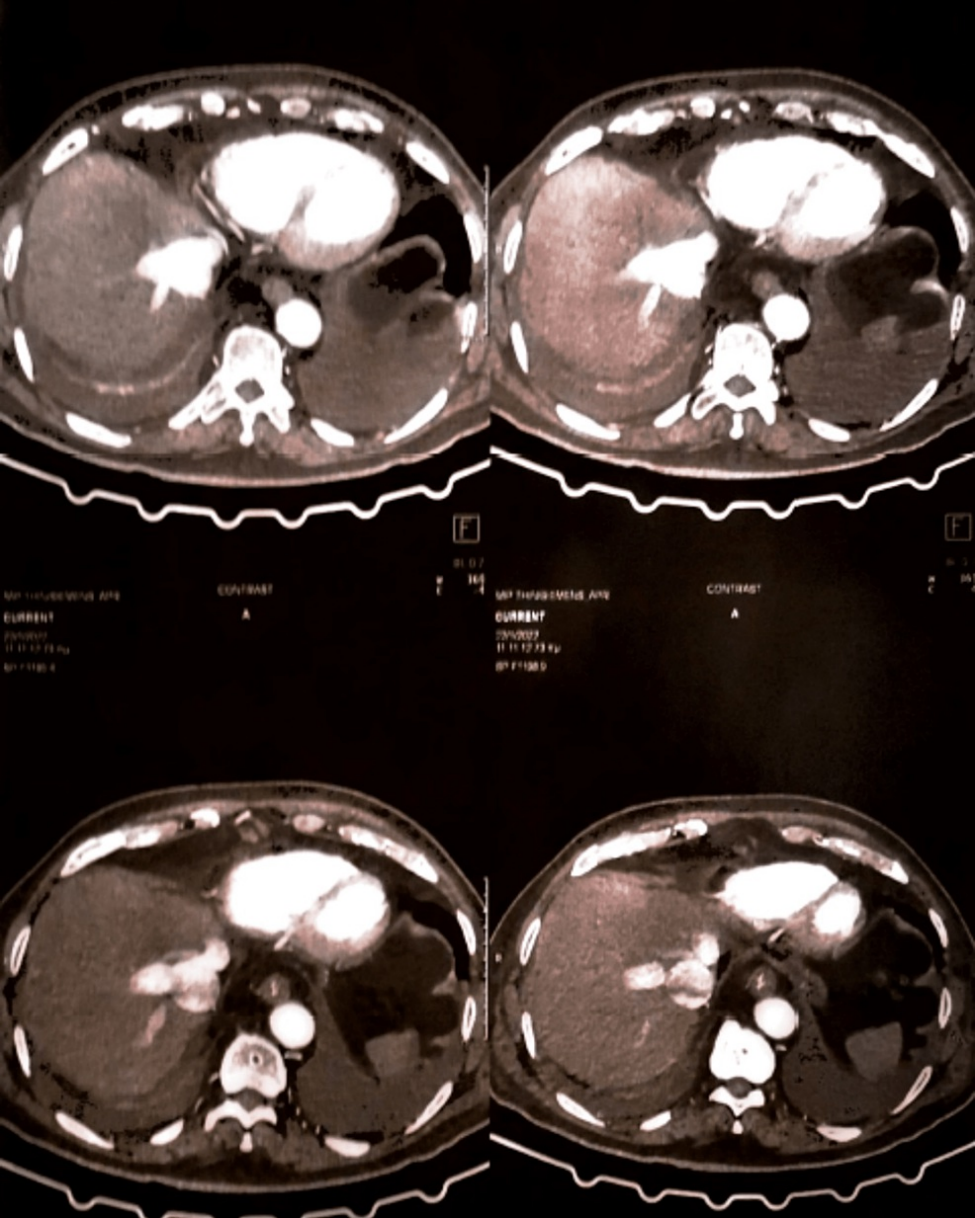
Computed tomography demonstrating mild liver enlargement, inferior vena cava dilatation, hepatic vein dilatation, and cardiac chamber enlargement.

A review of his vital sign chart did not reveal any episode of hypotension. An urgent abdominal CT demonstrated mild liver enlargement, inferior vena cava and hepatic vein dilatation, retrograde hepatic venous opacification shortly after intravenous contrast injection, and cardiac chamber enlargement (Figure 2, 3). The viral hepatitis panel was negative. On review of his medication chart remdesivir was considered the probable culprit and was discontinued two days after initiation. Given the CT and clinical findings, diuretic treatment was initiated. 

A steady decline of the transaminases and INR was observed 24 hours after the discontinuation of remdesivir, with the levels returning back nine days later. He remained throughout in good clinical condition, ambulatory, without any alteration regarding his level of consciousness.

His clinical course was complicated by nonsevere *Clostridium difficile* infection, which was treated successfully with oral metronidazole and vancomycin. He was discharged on day eleven, able to restart his normal daily activities without the need for home oxygen support.

## Discussion

According to the European Association for the Study of the Liver (EASL) acute liver injury is defined as the condition in which patients, without underlying chronic liver disease, develop an acute abnormality of liver blood tests and coagulopathy, but do not have any alteration to their level of consciousness [5]. In this case, the biochemical pattern of injury is hepatocellular (R=ALT/ALP≥5).

Drugs and toxins remain the main etiology of hepatocellular liver injury. Apart from drugs, the hepatocellular pattern of liver injury can be caused by viral, autoimmune, alcoholic, and hypoxic hepatitis. Given the history of the patient, the use of alcohol was two units per week. In addition, the vital sign chart did not reveal any episode of hypotension or respiratory deterioration, ruling out hypoxic hepatitis [6]. The viral hepatitis panel was negative, too. Autoimmune etiology of liver enzyme elevation has been excluded.

COVID-19 has been linked to elevated liver enzymes [7]. Liver injury has also been found to be more common in severe COVID-19 than in non-severe COVID-19 patients [8]. Elevated aminotransferase levels were indeed more frequent in intensive care unit inpatients as well as patients requiring ventilation [9]. Between the onset of COVID-19 symptoms and sudden ALT peak, there is a gap of five days, while liver enzymes were elevated only after 48 hours of remdesivir use. The known in vitro toxicity of remdesivir, the time relation, and automatic resolution after discontinuation of the drug are all aspects demonstrating a causal relation between elevated liver enzymes and remdesivir. Meanwhile, no other drug of his treatment can be related to hepatotoxicity.

In this case, a rise of more than ten times the upper normal limit of AST and ALT and coagulopathy with INR≥1.5 is observed. Using the US drug-induced liver injury network [5], the patient is classified as having moderate severity liver injury (Table 1) [5]. Goldman et al. and Wang et al. report a 1-2 ALT and AST elevation as the most common pattern in patients receiving remdesivir, demonstrating that, in most cases, elevated levels of liver enzymes do not progress to liver injury [10,11]. On the other hand, Leegwater et al. [12] presented a case of the potential interaction of remdesivir with P-glycoprotein inhibitors leading to acute liver failure (ALF). While Carothers et al. [13] presented two cases with two possible factors, each provoking ALF, amiodarone with remdesivir in the first case, and hypoxic hepatitis with remdesivir in the second case.

**Table 1:** DILI severity classification ALT: alanine transaminase, ALP: alkaline phosphatase, TBL: total bilirubin level, INR: international normalized ratio, DILI: drug-induced liver injury

Category	Severity	Description
1	Mild	Elevated ALT and/or ALP but TBL< 2.5mg/dl and INR<1.5
2	Moderate	Elevated ALT and/or ALP but TBL≥2.5 mg/dl or INR≥1.5
3	Moderate-severe	Elevated ALT, ALP, TBL, and/or INR and hospitalization or ongoing hospitalization prolonged due to DILI
4	Severe	Elevated ALT and/or ALP and TBL≥2.5mg/dl and at least one of the following criteria: hepatic failure (INR>1.5, ascites or encephalopathy); other organ failure due to DILI
5	Fatal	Death or liver transplantation due to DILI

**Figure 4:**
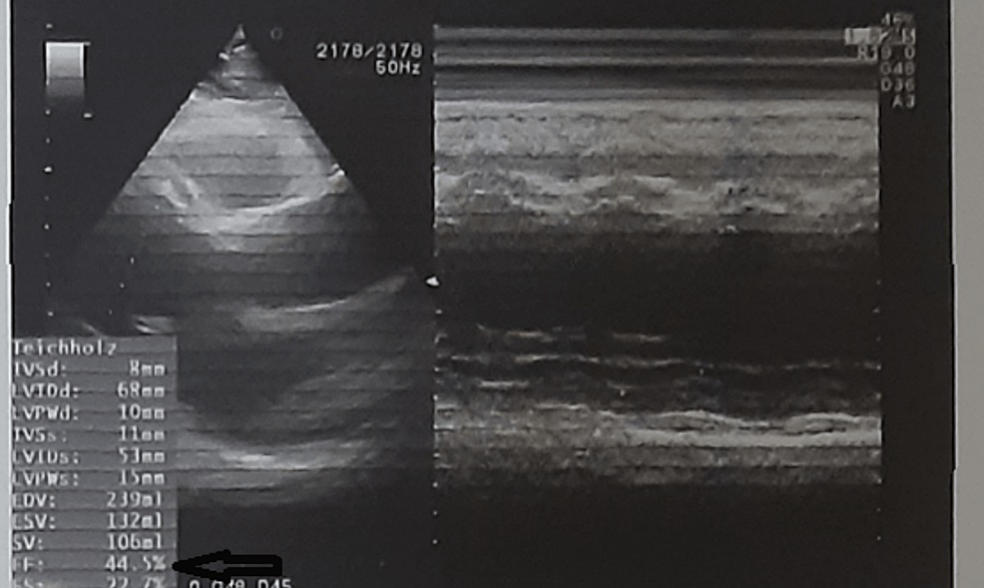
Echocardiography one week after discharge, demonstrating systolic dysfunction. Left ventricular ejection function 45%.

During the workup, computed tomography demonstrated inferior vena cava and hepatic vein dilatation, accompanied by slight enlargement of the liver and retrograde hepatic venous opacification of intravenous contrast material injection. All of the above are imaging findings of congestive hepatopathy [14]. The prevalence of liver congestion is high among patients with severe heart failure. Mitral stenosis, tricuspid regurgitation, constrictive pericarditis, and cor pulmonale are the most frequent causes of chronic heart failure provoking liver congestion. Taking into consideration the clinical signs of bilateral mild pedal edema, the need for diuretic treatment, and cardiac chamber enlargement depicted in CT, heart failure seems a possible underlying condition. Unfortunately, during his hospitalization, echocardiography was not possible. One week after the patient's discharge, echocardiography depicted severe systolic dysfunction (left ventricle ejection fraction=45%) (Figure 4).

Thus, undiagnosed pre-existing heart failure was possibly the etiology of congestive hepatopathy. Congestion and hypoperfusion of the liver result in impaired oxygen diffusion and hepatocyte atrophy leading to impaired metabolism of the liver [15, 16]. Chronic decrease in liver blood inflow and outflow results in chronic liver hypoxia. Due to a lack of hepatic venous valves, elevated venous pressure in congestive heart failure is transmitted to the sinusoidal bed. Sinusoids are small endothelium-lined capillaries, around small hepatic veins, with open pores increasing the permeability of the liver. Thus, sinusoidal congestion and peri-sinusoidal edema are caused by elevated venous pressure leading to decreased oxygen diffusion, atrophy of the hepatocytes, and impaired hepatocyte metabolism.

## Conclusions

As we mentioned, remdesivir is not able to provoke liver injury per se but only mild elevation of liver enzymes. Since recently, only a few cases of remdesivir-induced liver injury have been reported, and all of them were linked to either a combination with another drug or hypoxic hepatitis. We propose a possible synergistic act of remdesivir along with congestive hepatopathy as the etiology of acute liver injury in this patient.
